# Mating and post-copulation behavior in the tea leafhopper, *Empoasca onukii* (Hemiptera: Cicadellidae)

**DOI:** 10.3389/fpls.2023.1273718

**Published:** 2023-10-04

**Authors:** Yao Shan, Xiao-Sen Zhou, Xiao-Ming Cai, Zong-Xiu Luo, Zhao-Qun Li, Chun-Li Xiu, Zong-Mao Chen, Lei Bian

**Affiliations:** ^1^Tea Research Institute, Chinese Academy of Agricultural Science, Hangzhou, China; ^2^Key Laboratory of Biology, Genetics and Breeding of Special Economic Animals and Plants, Ministry of Agriculture and Rural Affairs, Hangzhou, China

**Keywords:** tea leafhopper, daily activity, vibrational communication, mating disruption, fertilized egg, copulation

## Abstract

The tea leafhopper, *Empoasca onukii*, relies on substrate-borne vibrations for sexual communication and is mainly controlled with chemical pesticides, which poses risks to the environment and food safety. Based on previous studies, we conducted a series of behavioral assays by simultaneous observation of vibration signals and movement to investigate the mating and post-copulation behavior of tea leafhoppers. During mating, the activity of *E. onukii* was restricted to dawn and dusk and concentrated on the sixth or seventh mature leaf below the tea bud. By comparing the time spent in locating females among different males, the timely reply of females was the key factor affecting mating success. *Empoasca onukii* females mated only once in their lives, while males could mate multiple times. Male rivalry behavior involved two distinct strategies. The rivals could send disruptive pulses to overlap the male calling signals, locate the courting males, and drive them away after contact. Some rivals could emit mating disruption signals (MDSs) to interrupt the ongoing identification duet and establish their own mating communication. Both identification and location duets could be interrupted by playback of MDSs, which is essential to create effective synthetic signals to disrupt mating communication of *E. onukii*. Our study clarified the spatial and temporal distribution of *E. onukii* in mating and the function of MDSs, which will be essential to develop future vibrational mating disruption techniques for *E. onukii* and its energy-efficient application in the field.

## 1 Introduction

Leafhoppers (Hemiptera: Auchenorrhyncha: Cicadellidae) are one of the most speciose groups of sap-sucking insects and are among the most important agricultural pests. These pests mainly rely on substrate-borne vibration for mating communication (Cocroft and Rodriguez, 2005), and species-specific vibrational signals have been described in a great number of leafhopper species, such as *Aphrodes makarovi* (Kuhelj and Virant-Doberlet, 2017), *Balclutha incisa* (Nuhardiyati and Bailey, 2005), *Empoasca vitis* (Nieri and Mazzoni, 2018) and *Scaphoideus titanus* (Mazzoni et al., 2009b). Importantly, exploitation of pest mating behavior and their vibrational signals revealed a new method of pest management (Eriksson, 2014), vibrational mating disruption (VMD), which has proven effective in preventing mating of the grapevine pest *S. titanus* (Polajnar et al., 2016; Zaffaroni-Caorsi et al., 2022).

Before development and application of VMD, a deeper understanding of the mating behavior of the target leafhopper species is necessary (Gordon and Krugner, 2019). The tea leafhopper, *Empoasca onukii*, has become a ubiquitous and predominant tea pest in East Asia over recent decades (Li et al., 2023). In previous studies, we briefly identified the vibration signals of *E. onukii* on a single leaf. Females emitted one signal type, and males emitted two signal types. Typically, *E. onukii* adults use sex- and species-specific vibration signals for mate identification, location, and courtship (Zhang et al., 2023). However, the previous results are far from enough for the development of VMD and there are many problems to be solved. First, the dominant frequency in the vibration signals of *E. onukii* will change dynamically with time, like the signals of the glassy-winged sharpshooter, *Homalodisca vitripennis* (Nieri et al., 2017). The frequency dynamics of a harmonic signal is also an important signal parameter. Second, there are significant differences in the parameters between the female signals emitted in response to the male calling signals and the male location signals, and female signals are probably two different types. Third, male *E. onukii* can easily find females on a single leaf, but this cannot replicate the localization process of *E. onukii* in a complex environment. In nature, where the environment is more complex, insects in low-density populations need to consume a lot of energy to search for and locate potential mates, so they may exhibit regular behaviors on complex hosts (Mazzoni et al., 2014). For example, the reproductive activity of *S. titanus* is restricted to a narrow time window (late afternoon/evening) to decrease the risks of predation and energy consumption (Mazzoni et al., 2009b). The planthopper, *Metcalfa pruinosa* (Virant-doberlet and Žežlina, 2007), gathers within small spatial distances, thus increasing the chances of mating. Therefore, observing the mating behavior of *E. onukii* on tea branches may provide more useful information for the field application of VMD to target this species (Polajnar et al., 2016). Most importantly, the mating competition behavior between males in the presence of multiple leafhoppers has not been studied. The competitive behavior and associated interference signals of target pests are the basis for investigating the feasibility of VMD techniques to control pests (Nieri and Mazzoni, 2018). Male competition is a common phenomenon in leafhopper mating behavior, during which the mating process can be delayed or interrupted by interference signals emitted by competitors (Nieri et al., 2017). Disruption of the mating process of target leafhoppers via rivalry signals has been reported in detail in *E. vitis* (Nieri and Mazzoni, 2019) and *S. titanus* (Eriksson, 2014). However, the competitive behavior of *E. onukii* during the mating process has not been reported so far.

Most research on mating communication that involves substrate-borne vibrational signals has focused on pre-copulation behavior, but vibrational signals are used during and after copulation in some insects, such as *Popillia japonica*, *Ozophora baranowskii*, and *Ozophora maculata* (Rodriguez, 2000; Rodríguez, 2015). Whether *E. onukii* emit vibration signals during and after copulation is still unknown.

As an environmentally friendly approach for pest management, VMD provides a practical option to control tea leafhoppers. In this study, we investigated the mating behavior of *E. onukii* during the courtship period on tea branches. Specifically, we examined the daily rhythm of calling activity, which can provide essential information for energy-efficient field application of VMD. We also investigated the spatial distribution of leafhoppers, which is necessary for identification of the intensity threshold of disruptive signals. Additionally, we observed sex-specific competitive behavior, the associated signals of which are the basis for the creation of disruptive signals in the development of VMD.

## 2 Methods

### 2.1 Plants

**Figure 1:**
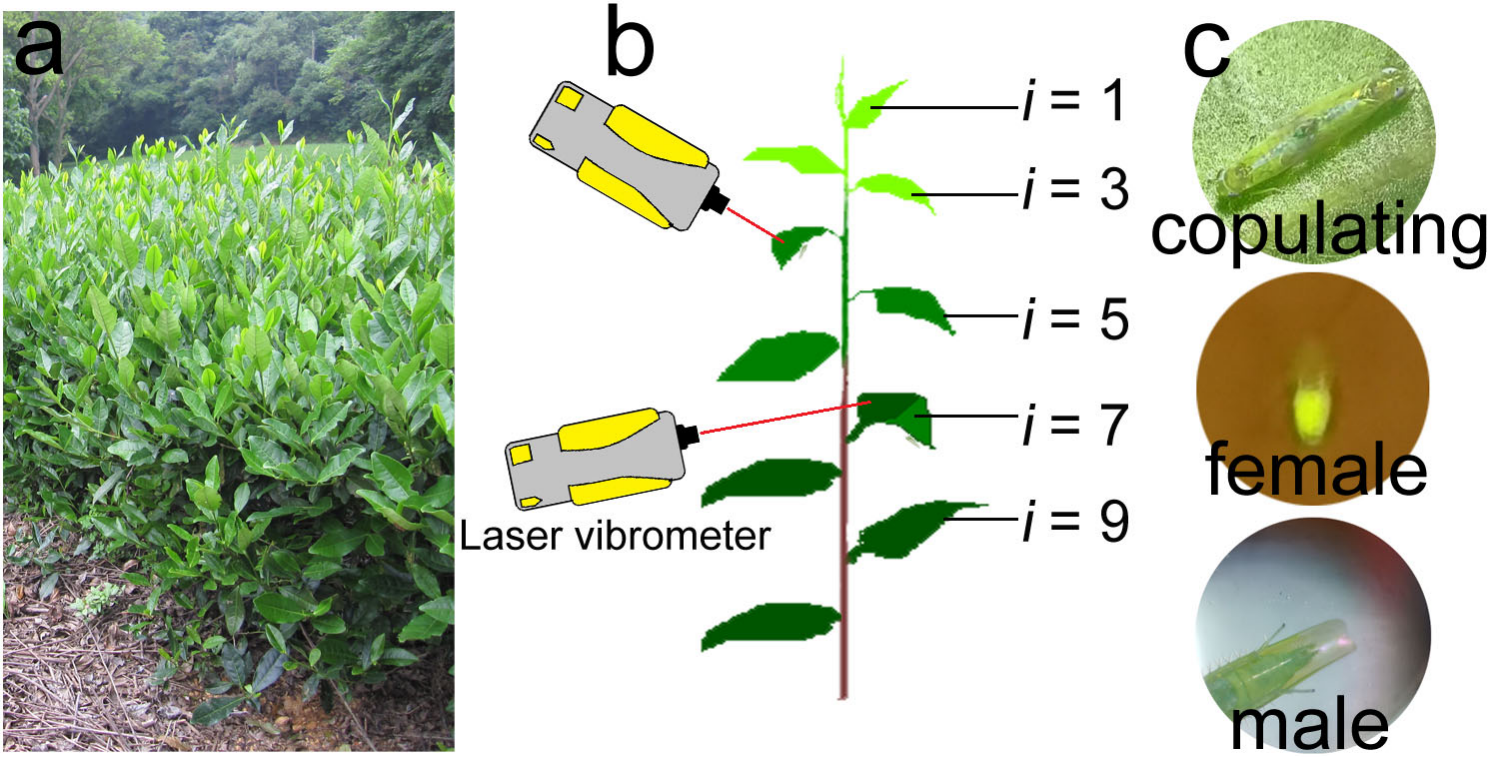
Appearance of tea plants, tea branches, and tea leafhoppers (*Empoasca onukii*). **(A)** Growth state of tea plants during the occurrence period of *E onukii*. **(B)** Set up used in the daily rhythm of copulation and post-copulation behavior experiment (Section 2.3.2) to record the mating behavior of *E onukii.* Tea branch showing different leaf stages of maturity (*i* = stages 1–10 from top to bottom). **(C)** A pair of copulating leafhoppers (top), a female with fertilized eggs under blue light (460 nm, middle) and a male’s genitals just after copulation (bottom).

Tea plants are farmed in cultivated shrub crops, and have a typical hierarchical structure (**Figure 1A**) in which the upper layers of branches are the production branches. At the top of each production branch is the tea bud, and below the bud are leaves of differing ages (i.e., the young leaves are closest to the buds, and the leaves increase in age with increasing distance from the bud). *Empoasca onukii* adults and nymphs prefer to feed on the mesophyll cells of the young leaves and stems (Bian et al., 2018a; Bian et al., 2020), so they are mostly distributed in the upper layer of production branches during feeding.

*Empoasca onukii* can complete their life cycle and reproduce on hydroponic tea production branches (Jin et al., 2012). The production branches used in this study (cultivar: Long Jing 43) were collected from the tea gardens at the Tea Research Institute, Chinese Academy of Agricultural Sciences (TRI, CAAS), Hangzhou, China (30.18 N, 120.09 E). All branches used for feeding or experiments were healthy and not affected by pests or disease. After being collected, the branches were immediately planted in sponges full of water. The sponges were covered with plastic wrap to retain moisture.

Branches with at least 10 leaves were used for observation of mating behavior in male-female pairs. From the top to the bottom of a tea branch, leaves below the bud were numbered according to increasing age (*i* = 1, 2 … 10, **Figure 1B**). The sixth or seventh leaf below the bud was used for individual feeding and other experiments, and the petiole was inserted into flower mud soaked in water to keep the leaf upright.

### 2.2 Insect rearing

Adult leafhoppers were collected in May and June in 2021 and 2022 from TRI, CAAS and then reared in a cage (60 × 60 × 60 cm) in the insectary with a stable environment (L14:D10, 25 ± 2°C, 75 ± 5% RH).

Using the feeding method outlined in Zhang et al. (2023), we obtained the virgin adults used for testing from the second and subsequent generations. After emergence, the adults were reared individually with one leaf in fruit fly tubes (24 × 95 mm), which were marked with the date of emergence and sex. Seven-day-old virgin *E. onukii* adults were used in the subsequent experiments. The leaves were replaced every 3 d and all leafhoppers tested were used only once. We observed the mating behaviors, daily rhythm of copulation and egg-laying behavior of the leafhoppers.

### 2.3 Recording vibrational signals and behavior

The acquisition and parameter analysis of specific signals will provide the databases for creating the disruptive signals that can effectively block the mating process of target pests (Mazzoni et al., 2019). Vibrational signal recordings were made in an anechoic room at TRI, CAAS with the same environment as the insectary. The laser vibrometer (PDV-100 or VGO-200, Polytec GmbH, Waldbronn, Germany) focused on a reflecting sticker was used to detect the vibrational signals from the leaf lamina. Before signal acquisition, the laser vibrometers were calibrated using the accelerometer calibrator (Type 4294, Brüel and Kjær, Virum, Denmark). Signals from the laser vibrometer (PDV-100) were digitized with a 48-kHz sample rate and 16-bit depth and transmitted to a computer via the digital audio interface (U24 XL; ESI Audiotechnik GmbH, Leonberg, Germany). Signals were displayed in real-time and saved in WAVE format with Cool Edit Pro version 2.1 (Syntrillium Software). Signals from the laser vibrometer (VGO-200) were transmitted via the generator module (LAN-XI 3160, Brüel and Kjær) and saved using BK Connect^®^ software (Brüel and Kjær).

The characteristics of pest mating and competitive behavior need to be determined to develop methods suitable for VMD field applications, including the effective range of disruptive signals and their appropriate playback time range (Mazzoni et al., 2019). The observation of daily rhythm of calling activity (Section 2.3.1) and copulation (Section 2.3.3), post-copulation behavior (Section 2.3.3), and male rivalry behavior and playback of vibrational signals (Section 2.3.4) were conducted on a single leaf, with the leaf and tea leafhoppers placed in a plastic 6-cm diameter Petri dish. The behavior of *E. onukii* was recorded and saved via two webcams (C1000e, Logitech, Lausanne, Switzerland) on each side of the leaf, which enabled us to observe the vibrational signals and target behavior simultaneously. The observations of mating behavior of a male-female pair (Section 2.3.2) and egg-laying behavior of females (Section 2.3.3) were carried out on a production branch, with the branch and tea leafhoppers placed in a rectangular plexiglass box (20 × 20 × 35 cm) with several small holes (diameter 1 mm) on each side to ensure ventilation. The mating behavior of *E. onukii* was recorded with three webcams, one focused on the female leaf, one on the male leaf, and the remaining one focused on the stem between the two leaves. Reflective stickers were placed on each leaf of the branch. All following experiments were conducted in the same environment as the insectary.

#### 2.3.1 Daily rhythm of calling activity

Calling activity was tested to evaluate the mating peak of *E. onukii*. We used the laser vibrometers to continuously monitor the number of calling signals emitted by a male or female leafhopper on a single leaf every hour. This experiment was repeated 10 times using a new pair each time.

#### 2.3.2 Mating behavior

**Figure 2:**
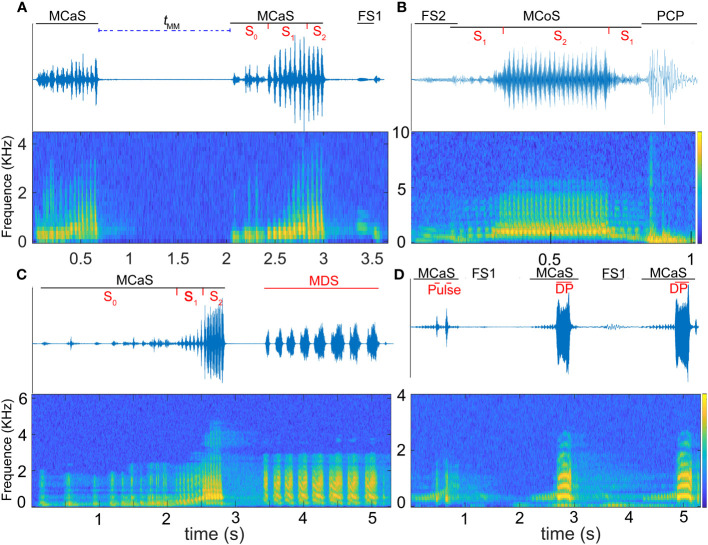
Oscillogram (above) and spectrogram (below) of *Empoasca onukii* vibrational signals. **(A)** Identification duet formed by two consecutive male calling signals (MCaSs) and one female signal response to the MCaS (FS1), t_MM_ is the interval from the end of an MCaS to the beginning of the next neighboring MCaS. **(B)** Courtship duet before copulation formed by one male courtship signal (MCoS) and one female signal response to the MCoS (FS2), and a pre-copulation pulse (PCP) emitted when the male jumps up and touches the female. **(C)** Competing duets formed by one MCaS and one mating disruption signal (MDS). **(D)** An on-going identification duet; the rival sends disruptive pulses (DPs) to overlap the MCaSs. S_0_ to S_2_ in **(A–C)** represent different sections of the MCaS.

Observation of mating behavior of a male-female pair on tea branches was conducted to determine the spatial distribution of both sexes, the probability of specific behaviors occurring in each stage, and the time spent by males to locate females. This trial was repeated 203 times, and in each trial a new production branch was used. Before testing, the laser vibrometer and webcams were turned on, a female leafhopper was placed into the plexiglass box, and then the laser vibrometer (PDV-100) and one webcam were focused on the leaf where the female flew to and stayed. After that, a male leafhopper was placed into the box. When the male flew to a leaf and stayed on it, we used the laser vibrometer (VGO-200) and one webcam to record the male calling signal (MCaS) and accompanying behavior in real-time (**Figure 2B**). The number (*n_i_*) of female and male leafhoppers on each leaf (*i*) that emitted their first signal was recorded. At the same time, webcams were used to record the movement of the male and time spent on the branch. In 203 trials, we counted the number of pairs that completed the identification stage and pairs that successfully copulated. For trials in which successful mating occurred, we then counted the number of distinct behaviors of males in the location stage. For trials in which the pairs failed to mate, we identified the causes and then counted the number of times in different stages at which the failure occurred. The trials were stopped if the male and female copulated or the location time was more than 2 h.

#### 2.3.3 Daily rhythm of copulation and post-copulation behavior

The daily rhythm of copulation was tested to evaluate the copulating peak of *E. onukii*. We randomly selected pairs of leafhoppers (*N* = 60) every day and reared them in the fly tubes. The number of copulating leafhoppers per hour was continuously observed for 24 h. The beginning of copulation was regarded as when the genitals of male and female leafhoppers joined together and did not separate within 1 min (**Figure 1C**). Pairs that failed to copulate within 24 h were not included in the statistical analyses. This experiment was repeated five times.

Observation of post-copulation behavior was conducted to determine whether *E. onukii* emit vibration signals during and after the first copulation. We selected 26 copulating pairs to record the duration and vibration signals during the first copulation using webcams and a laser vibrometer (PDV-100). After copulation, the status of the male and female was observed, and then they were transferred to two new tubes. Then a 7-day-old virgin female was added into the male tube to observe whether the male could mate again, and the interval between the first and second copulation and the duration of the second copulation were measured. After the second copulation, this procedure was repeated until the male died.

Females prefer to lay eggs on young stems and leaves, and the eggs will fluoresce under 460-nm blue light (Yao et al., 2020). A mated female (*N* = 30) was randomly selected and transferred to a production branch in the plexiglass box. We checked the number of eggs laid every 6 h during the first day after copulation and every other day thereafter. Then the gestation period (the interval from copulation to the first egg laying), egg-laying period (the interval from the first to the last egg laying) and total number of eggs were determined. After the female stopped laying eggs, a 7-day-old virgin male was added to observe whether the female could mate repeatedly.

#### 2.3.4 Male rivalry behavior

Male rivalry behavior was determined to obtain competitive signals, which were then played back to assess their effects on the mating behavior of a pair of leafhoppers, and would be used as the basic disruptive signals for the future development of VMD. Virgin males were tested in four contexts: two males (*N* = 15), four males (*N* = 15), two males and one female (*N* = 29), and two males and two females (*N* = 16). Recording with the laser vibrometer (VGO-200) and two webcams started when all leafhoppers were on the leaf surface, and ended when copulation began or after 2 h.

Playback of two types of competitive signals, including mating disruption signals (MDSs) and disruptive pulses (DPs), was conducted with a mini-shaker (Type 4810, Brüel and Kjær). The mini-shaker, controlled by a computer and BK Connect^®^ software, was used to send vibrational signals to the leaf, where a male-female leafhopper pair was located. A conical rod attached to the mini-shaker was in contact with the lower lamina of the leaf. The intensity of the playback signals was adjusted to the same level as the natural intra-specific signals emitted from male leafhoppers. Based on the parameters of the vibrational signals, the duration of MDS playback was 4.48 s (composed of 13 pulse trains). Section 1 (S1) in the MCaS and MCoS had a mean length of 0.283 and 0.3 s, respectively (Zhang et al., 2023). During playback, DPs repeated every 0.3 s, i.e., frequently enough to ensure overlap with any potential S1 in the MCaS or MCoS. We played back one MDS and a series of DPs to disrupt the identification duet and location duet, respectively. For MDS playback, one MDS was played immediately after the male emitted the second MCaS in an identification duet. During the location process, we played back one MDS to disrupt the location duet after the first male courtship signal (MCoS). For DP playback, a series of DPs was played immediately after the male emitted the second MCaS in an identification duet and after the second MCoS in a location duet. The DP playback stopped if copulation began or more than 10 min had elapsed. After the playback, we observed the behavior of the leafhoppers and calculated the interval between two neighboring MCaSs. If the male re-entered the call-fly stage, the playback was considered to have successfully blocked the duet. Each playback experiment was repeated 15 times.

### 2.4 Terminology and statistical analyses

#### 2.4.1 Analysis of signal characterization

Vibrational signals were identified and named based on a combination of signal structure, behavioral context, and previous studies (Mazzoni et al., 2009a; Nieri and Mazzoni, 2019; Zhang et al., 2023): male calling signal (MCaS), male courtship signal (MCoS), female signal response to the MCaS (FS1, **Figure 2A**), female signal response to the MCoS (FS2, **Figure 2B**), pre-copulation pulse (PCP, **Figure 2B**) emitted by a male when it touched a female, mating disruption signal (MDS), and disruptive pulse (DP).

Spectral and temporal analyses were performed using Adobe Audition 2021 (Adobe Systems Incorporated, San Jose, CA, USA) using Fast Fourier Transform (FFT) type Hann, with a window length of 8192 samples and 75% overlap. The following parameters, when applicable, were measured for each signal: signal duration, pulse number (*No*. pulses), pulse repetition time (*PRT*), dominant frequency (*Df*), and the intensity of the FS. The MCaS consisted of three sections, S0, S1, and S2 (**Figures 2A, C**). The *Df* of S0 and S2 were basically consistent in the pulse train, and the *Df* of S1 and the FS were dynamically changed. With reference to the method of Nieri et al. (2017), we measured the *Df* of each pulse in S1, *Df* at the beginning (*Df*_b_), three internal points, and the end (*Df*_e_) of the FS. With the time point at which the *Df* was measured as the independent variable and *Df* as the dependent variable, linear fitting was carried out, and the slope of the curve was regarded as the modulation rate (*MR*).

Each behavioral experiment resulted in a signal sample and a video sample. Five signal samples were randomly selected from the male-female pair experiments during which successful mating occurred. Then, we randomly selected one MCaS, FS1, and FS2 from each signal sample. Five signal samples containing MDSs and five signal samples containing DPs were selected from the male rivalry experiment, and then one MDS and DP were randomly selected from each signal sample to analyze the signal parameters. The parameter difference between FS1 and FS2 from a signal sample was first analyzed with a paired *t*-test using SPSS 19.0 (IBM Corp., Armonk, NY, US) to evaluate whether FS1 and FS2 were two distinct female signals. Then the parameter differences among the MCaSs, FS1s, FS2s, MDSs, or DPs from different samples were analyzed using one-way ANOVA to assess whether there were differences in signals triggered by different individuals. Finally, we performed discriminant analysis on different signals to determine whether signals could be distinguished based on temporal (duration/*PRT*) and spectral (*Df*_b_, *Df*_e_, and *MR*) profiles.

#### 2.4.2 Analysis of behavioral parameters

In Section 2.3.1, the number of MCaSs or FSs spontaneously emitted by a male or female per hour were defined as the calling activity (*A*) of *E. onukii*. The number of copulating leafhoppers per hour was defined as copulating activity (*C*) (Section 2.3.3). The calling and copulating rhythm were analyzed with time as the independent variable and *A* and *C* as dependent variables, and the peaks of *A* and *C* were then evaluated with nonlinear regression (log normal, 3 parameter) using SigmaPlot (version 11.0, Systat Software Inc., San Jose, CA, USA).

We recorded the number of leafhoppers (*n_i_*, Section 2.3.2) on each leaf (*i*, Section 2.1) that emitted their first signal, and then determined the spatial distribution of males and females on tea branches using nonlinear regression (log normal, 3 parameter) with *i* as the independent variable and *n_i_* as the dependent variable, respectively.

In the mating behavior experiments (Section 2.3.2), we recorded the movement of the male on the branch and the time spent in the location stage, which was defined as *t*_L_. At the beginning of mating, the number of leaves (*n*_m-f_) between the leaves on which the male and female were located was the absolute value of *i*_male_ minus *i*_female_. During the location process, sometimes the male paused and restarted the identification process, which we defined as a location cycle. We defined the number of location cycles as *m*_L_. Finally, the Pearson correlation coefficient between three variables (*n*_m-f_, *t*_L_ and *m*_L_) was calculated to evaluate whether the spatial distribution of males and females on the branch could affect the location time.

Five signal samples containing MDSs and five signal samples containing DPs were randomly selected for the following analysis. We defined the interval from the end of an MCaS to the beginning of the next MCaS as *t*_MM_, including *t*_MM-FS_ (an FS1 occurred between two MCaSs), *t*_MM-MDS_ (an MDS occurred between two MCaSs), *t*_MM-DP_ (a DP overlapped the MCaS), and blank control *t*_MM_ (no signal occurred between two MCaSs). In the initial stage of mating communication, the male would send two or three MCaSs in a row, and the value of *t*_MM_ was significantly extended in the presence of a female response or the interference of other males. By comparing *t*_MM_ values of different types from the same signal sample using a paired *t*-test (SPSS 19.0), the delay effect of the FS, MDS, or DP on mating communication could be calculated. We played back the MDS or DP (Section 2.3.5) to disrupt the identification duets and location duets. The differences between *t*_MM_ and *t*_MM-MDS_ or *t*_MM-DP_ were analyzed with a paired *t*-test to calculate the effect of MDS or DP playback on the identification duets and location duets, respectively.

## 3 Results

### 3.1 Signal characterization and discrimination

FS had a typical harmonic structure (**Figure 2**), and the *Df* decreased with time (*MR*_FS_ < 0). The significant differences between the signal parameters of FS1 and FS2 (**Table S1**), including signal dominant frequency (*Df*_b_ and *Df*_e_, *P* < 0.05), duration (*t* = 7.906, *df* = 8, *P* < 0.001), intensity (*t* = 6.203, *df* = 4, *P* = 0.003), and *MR*_FS_ (*t* = 6.097, *df* = 4.404, *P* = 0.003), indicated that FS1 and FS2 were two different types of signals. There were no significant differences in FS1 signal parameters among different individuals (*P* > 0.05), but there were significant differences in FS2 signal duration (*F* = 5.957, *df*_1_ = 4, *df*_2_ = 20, *P* < 0.05) and *MR*_FS_ (*F* = 21.893, *df*_1_ = 4, *df*_2_ = 20, *P* < 0.05), indicating that FS2 emitted from different individuals varied greatly (**Table 1**).

**Table 1:** Spectral and temporal parameters of *Empoasca onukii* female and male vibrational signals.

	*Df*_b_ (Hz)	*Df*_e_ (Hz)	*MR* (Hz/ms)	Duration/*PTR* (s)	*No*. pulses
FS1	553.99 ± 52.6*	244.7 ± 57.19	−1.44 ± 0.44	0.23 ± 0.04	1
FS2	520.71 ± 52.81*	352.36 ± 62.05*	−1.55 ± 0.85*	0.14 ± 0.027*	1
DP	382.53 ± 62.72*		–	0.37 ± 0.094*	1
MCaS-S0	310.28 ± 44.59		0	0.054 ± 0.028	6 ± 4.16
MCaS-S1	369.55 ± 63.05	538.65 ± 157.12	0.95 ± 0.36	0.047 ± 0.013	6.67 ± 1.5
MCaS-S2	709.55 ± 312.79		0	0.035 ± 0.007	6.56 ± 1.42
MDS	448 ± 24.66	266.32 ± 11.9	−1.18 ± 0.52	0.24 ± 0.1	12.6 ± 5.17*

FS1, female signal response to the MCaS; FS2, female signal response to the male courtship signal; DP, disruptive pulse; MCaS, male calling signal; S0, S1, and S2 are the first, second and third section of the MCaS, respectively. MDS, mating disruption signal of rivals. Df_b_, starting dominant frequency. Df_e_, ending dominant frequency. MR, modulation rate determining the Df rate of increase/decrease within a signal. PRT, pulse repetition time. * indicates a significant difference among the signal parameters from different individuals (one-way ANOVA, P < 0.05).

-, MR of DP cannot be calculated because its dominant frequency changes in an inverted U-shape.

The MCaS consisted of three sections, S0, S1 and S2 (**Figures 2A, C**). The *Df* of MCaS-S1 increased with time (*MR*_MCaS-S1_ > 0), while S0 and S2 remained constant (**Table 1**). There was no significant difference in MCaS signal parameters among different individuals (*P* > 0.05). The PCP was emitted by the male when it touched the female (*Df* = 245.48 ± 22.99 Hz, duration = 0.15 ± 0.042 s; **Figure 2B**).

In the male rivalry behavior, two types of interference signals were identified, including the MDS and DP (**Figures 2C, D**). The MDS was composed of a series of pulses with a duration of 4.15 ± 1.84 s. The *Df* of a single pulse decreased over time (*MR*_MDS_ < 0), and the *PRT* of the pulses in the MDS was inconsistent, the first pulse being shorter. The number of pulses varied significantly among different individuals (*F* = 13.42, *df*_1_ = 4, *df*_2_ = 20, *P* < 0.001). The DP was a monopulse signal with a clear harmonic structure, and its *Df* changed in an inverted U-shape, first increasing and then decreasing. The *Df* (*F* = 286.74, *df*_1_ = 4, *df*_2_ = 20, *P* < 0.05) and duration (*F* = 17.21, *df*_1_ = 4, *df*_2_ = 20, *P* < 0.05) were significantly different among individuals.

**Figure 3:**
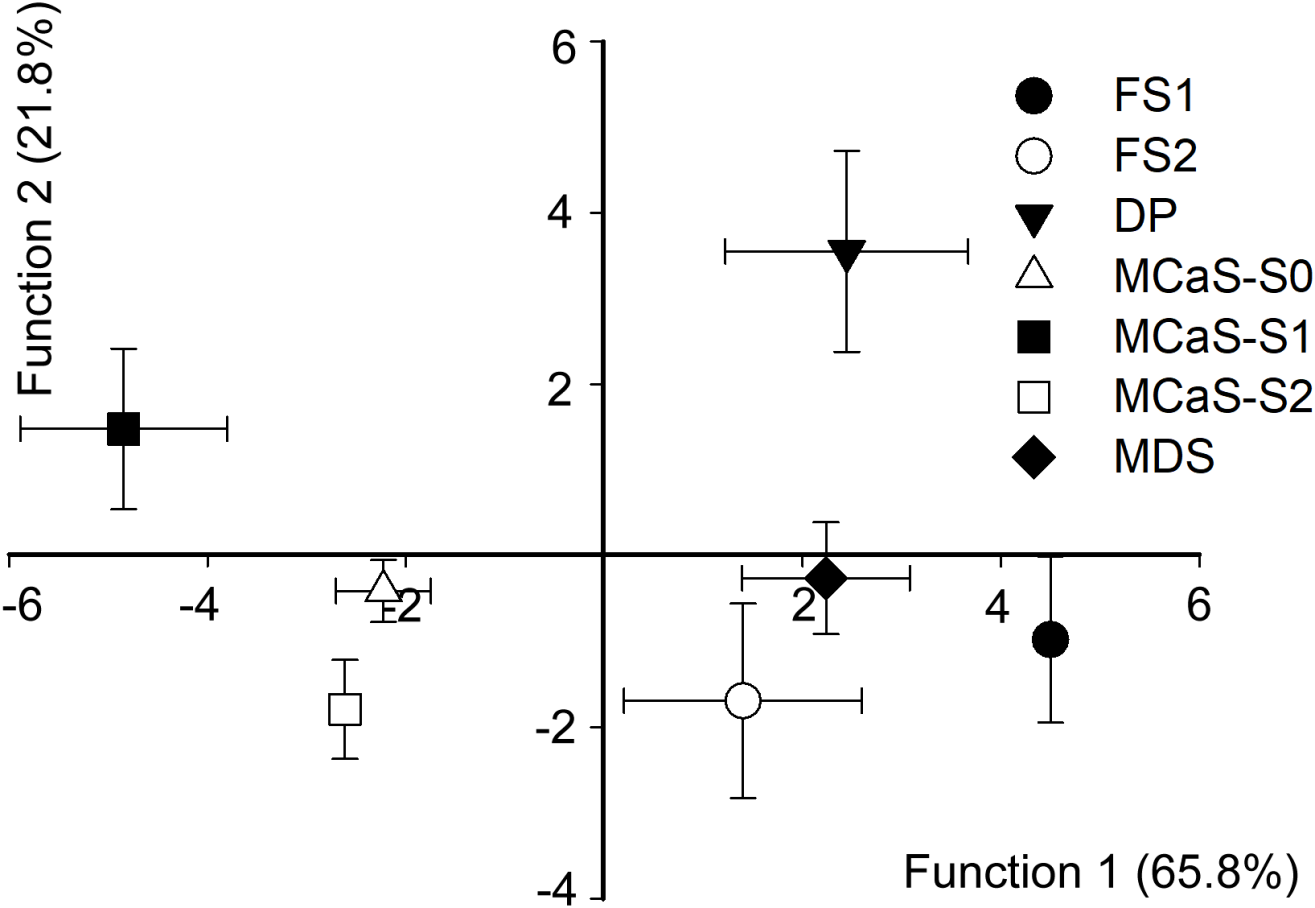
Combined-groups plot of *Empoasca onukii* vibrational signals. Functions 1 and 2 were derived via discriminant analysis of signal duration (or pulse repetition time), starting dominant frequency (*Df*_b_), ending dominant frequency (*Df*_e_) and modulation rate (*MR*). Functions 1 and 2 account for 65.8% and 21.5% of variance, respectively. Discrimination between female signal response to male courtship signal (FS2) and pulses in the mating disruption signal (MDS) was minimal. FS1, female signal response to male calling signal (MCaS); DP, disruptive pulse; MCaS-S0, MCaS-S1 and MCaS-S2 represent the first three sections of the MCaS (see **Figure 2**).

Discriminant analysis indicated that temporal and spectral parameters played a decisive role in determining signal specificity. The overall accuracy of discrimination was high (93.9% of the signals were correctly classified). The first two discriminant functions explained 87.6% of the variance (function 1 = 65.8%, canonical correlation = 0.963, Wilks’ *λ* = 0.003, *χ*^2^ = 153.06, *P* < 0.001; function 2 = 21.8%, canonical correlation = 0.81, Wilks’ *λ* = 0.9, *χ*^2^ = 83.56, P < 0.001). The standardized canonical discriminant function coefficients are reported in **Table S2**. The plot from the discriminant analysis (**Figure 3**) shows that all signals involved in the call-fly and identification stage, namely MCaS, FS1, FS2, MDS, and DP, could be easily distinguished from each other (accuracy > 60%, **Table S3**).

### 3.2 Spatial distribution and daily rhythm of mating behavior

**Figure 4:**
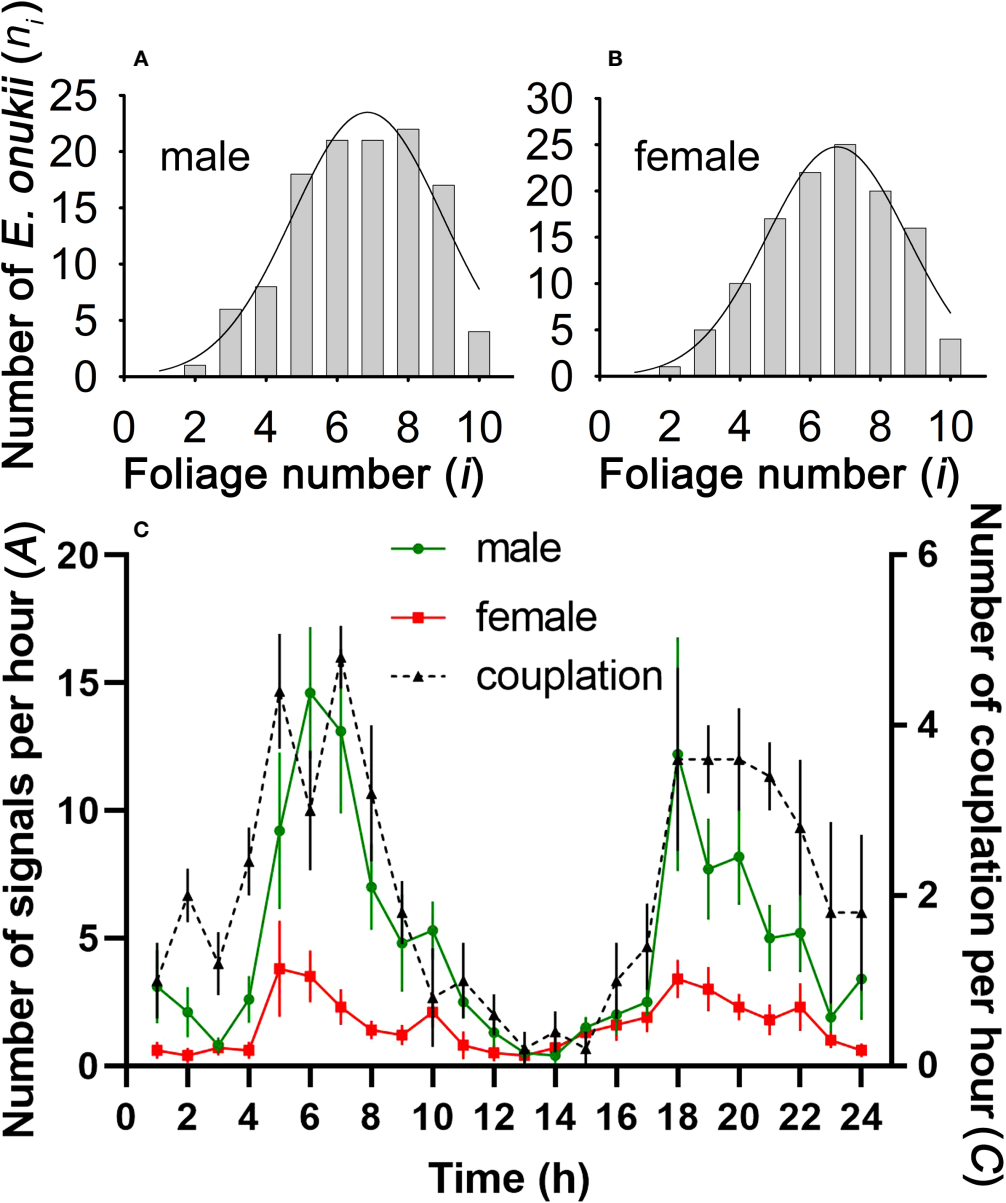
Spatial and temporal distribution of mating *Empoasca onukii*. **(A, B)** Distribution characteristics of male and female *E onukii* on tea leaves at different stages of maturity (*i* = stages 1–10) (see **Figure 1**). The fitting curve (log normal, 3 parameter) was obtained using SigmaPlot (version 11.0). **(C)** Vibrational and copulatory activities of *E onukii* at different times of day. *A* is the number of MCaSs or FSs spontaneously emitted by a male or female per hour, and *C* is the number of copulating leafhoppers per hour.

In the mating stage, *E. onukii* adults (both female and male) preferred to send vibration signals on mature leaves, mostly distributed on the sixth to eighth leaves below the bud. The peak distribution position of males was the 6.855^th^ leaf below the bud (*N* = 120, *R*^2^ = 0.941, **Figure 4A**), and for females it was the 6.769^th^ leaf below the bud (*N* = 120, *R*^2^ = 0.948, **Figure 4B**).

Both male and female leafhoppers spontaneously sent mating signals throughout the day and preferred to send signals at dawn and dusk (**Figure 4C**). The calling activity (*A*) of males was 4.87 ± 0.43 times/h, which was higher than that of females (1.59 ± 0.15 times/h, *t* = 4.93, *df* = 23, *P* < 0.001). The peak of male calling activity was 06:45 (*N* =10, *R*^2^ = 0.899, *P* < 0.01), and 19:21 (*N* = 10, *R*^2^ = 0.709). Females were active at 06:38 (*N* =10, *R*^2^ = 0.696, *P* < 0.05) and 18:55 (*N* = 10, *R*^2^ = 0.855). The number of copulating leafhoppers per hour (*C*) also revealed the time windows for mating activity (**Figure 4C**), which peaked at 06:07 (*N* = 5, *R*^2^ = 0.808, *P* < 0.01) and 20:11 (*N* = 5, *R*^2^ = 0.941, *P* < 0.01).

### 3.3 Description of mating behavior

**Figure 5:**
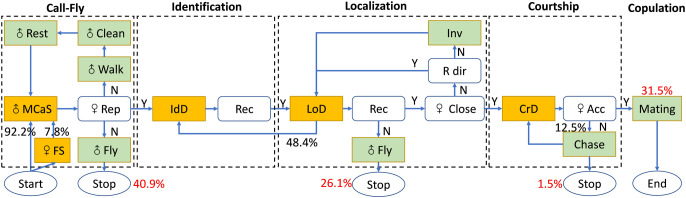
Flow chart with the behavioral steps of male *Empoasca onukii* while searching for a female on a tea branch. The black numbers describe the transition probabilities between behaviors that constitute the process of copulation formation. The red numbers represent the ratio of the total number of successful pairs and unsuccessful pairs at each stage. The signals (MCaS, FS, IdD, LoD, and CrD) of *E. onukii* are shown in yellow boxes and the behavior (e.g., walk, fly) of males at each stage is shown in light green boxes. MCaS, male calling signal; FS, female signal; IdD, identification duet; LoD, localization duet; CrD, courtship duet; ♀ rep, female reply; Rec, recognition; ♀ close, distance between male and female does not exceed 5 mm; ♀ Acc, female accepts copulation with male; R dir, right direction; Inv, inversion of direction. The flow chart was designed based on Polajnar et al. (2014).

Like other leafhoppers, the mating behavior of *E. onukii* adults was conservative and was divided into five stages: male call-fly, female identification, male location, courtship and copulation (**Figure 5**). In all trials (*N* = 203), 120 pairs sent mating signals and completed the identification stage, and only 31.5% (64/203) pairs started copulation within 2 h. In the 139 unsuccessful mating trials, 40.9% (83/203) females did not reply to MCaSs within 2 h, 26.1% (53/203) females did not reply to MCoSs in the location stage and three trials ended in the pre-copulation stage, which indicated that the female’s prompt response was the key factor affecting the success of mating.

In the call-fly stage, 92.2% of males (59/64) emitted MCaSs, and only five females spontaneously emitted FSs. Without female replies, the male would fly to other leaves after emitting two or three MCaSs in a row, with a *t*_MM_ of 0.62 ± 0.14 s. If the female replied, the male and female formed an identification duet, and the *t*_MM-FS_ increased significantly (1.01 ± 0.31 s, *t* = 7.18, *df* = 4, *P* < 0.001). Mostly, the male would send MCaSs again two or three times during the identification stage, and then entered the location stage. Interestingly, 28.13% (18/64) of males that emitted MCaSs on a leaf immediately moved to a nearby stem to wait for a response from a potential female, even if both were on the same leaf.

The location stage is the key stage of mating behavior, and the time spent in the location stage (*t*_L_ = 10.83 ± 19.98 min) could account for more than 90% of the whole mating process. The male emitted the MCoS and moved at the same time, and could complete the location stage within a minimum 0.27 min. Sometimes the males misjudged the direction of the female during location, and even moved to the wrong leaf. Sometimes the male paused and re-started from the identification stage, in which case the location could take multiple location cycles, and the longest *t*_L_ was 110.47 min. Of the 64 trials, 51.56% (33/64) of males found females within one location cycle (*m*_L_ ≤ 1), which took 1.56 ± 0.99 min; 29.69% of males (19/64) took two to six location cycles to locate the female, which took 5.37 ± 3.62 min. When there were more than six location cycles, the value of *t*_L_ was 44.97 ± 25.62 min. In the location process, the value of *n*_m-f_ did not affect the time spent in the location stage. The value of *m*_L_ was positively correlated with the value of *t*_L_ (correlation coefficient = 0.92, *P* < 0.001). When 6 ≥ *n*_L_ ≥ 2, the interval between location cycles ranged from 0.05 to 4.17 min. Most females remained in the same position during the location stage, and in three trials the females flew to other leaves, but still maintained the location duet.

When the male and female insects were on the same leaf and the space was less than approximately 5 mm, the male would enter the courtship stage and then jumped up to try to copulate with the female, emitting one or two PCPs (**Figure 2B**). However, 12.5% of the females (8/64) rejected copulation at this pre-copulation stage and moved, flying to other leaves, while the males chased the females, courted again and finally copulated. As mentioned above, 1.5% (3/203) of trials of this type (female rejection at the pre-copulation stage) resulted in mating failure.

### 3.4 Description of copulation and egg-laying

**Figure 6:**
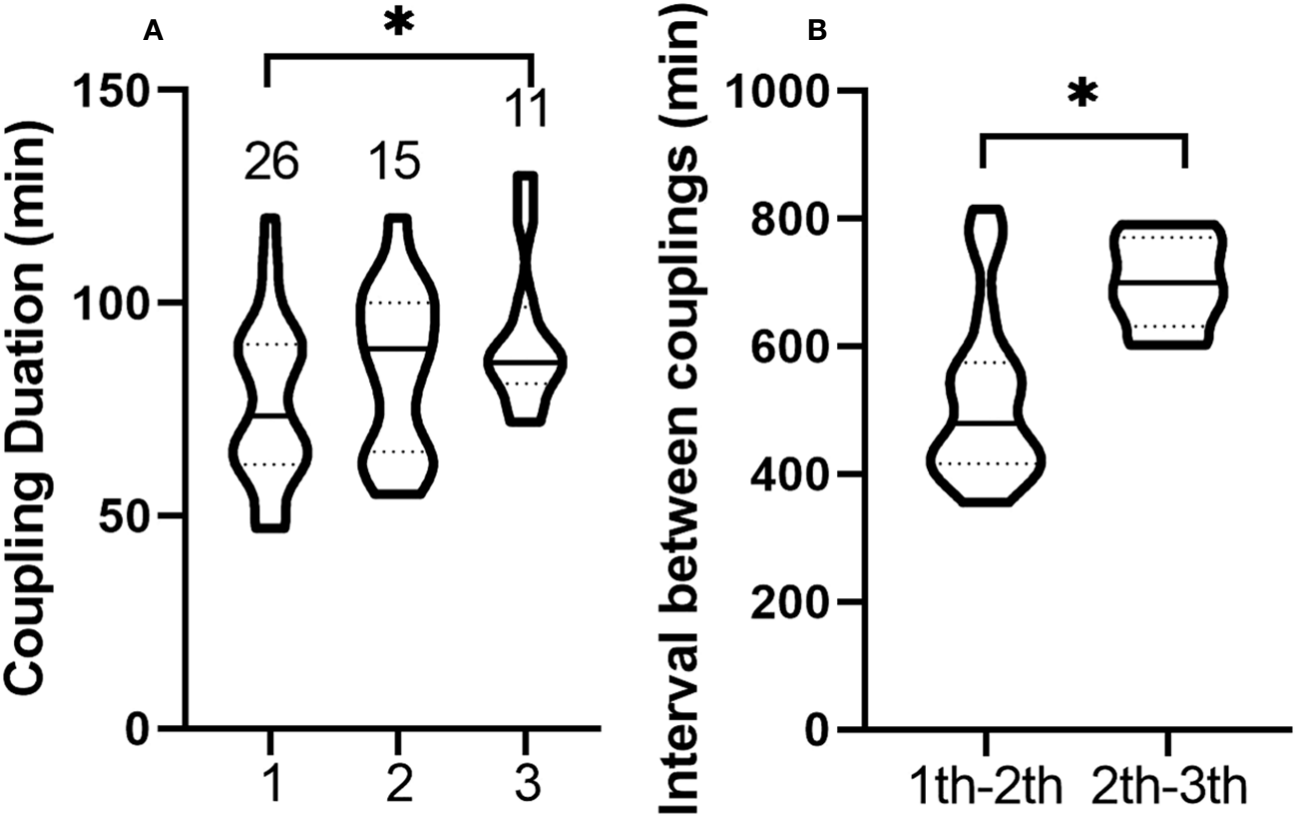
Violin plots of duration and interval of multiple copulations of *Empoasca onukii* males. **(A)** Duration of the first, second, and third copulation of males. The numbers above each plot represents the number of samples analyzed. **(B)** Interval between the first and second copulation of males, and the interval between the second and third copulation of males. The solid line in each plot represents the median and the dashed line represents the quartile. * indicates there is a significant difference (*P* < 0.05, unpaired *t*-test).

Neither male nor female leafhoppers emitted vibration signals when copulating, and the duration of copulation ranged from 47 to 130 min. Males could mate up to three times. Of the 26 males, 57.7% (15/26) mated twice and 42.3% (11/26) mated three times. After copulation, the genitals of the males were forked (**Figure 1C**) and closed within 1 min. As the number of copulations increased, the copulation duration was extended, and the duration of the third copulation was significantly longer than that of the first one (**Table S1**, *t* = 2.248, *df* = 35, *P* = 0.031, **Figure 6A**). After copulation, males needed an interval of 5.92–13.58 h before the next mating. The interval between two copulations increased significantly (*t* = 2.881, *df* = 24, *P* = 0.008, **Figure 6B**). The male did not die immediately after the third copulation and could still live for 3 to 7 d.

If the eggs were successfully fertilized, the female would no longer mate with other males and started to lay eggs after 1.9 ± 2.01 d. If the eggs were not successfully fertilized, females would mate again with other males. The duration of the egg-laying period was 10 to 28 d (23.32 ± 4.38), and the number of eggs laid was 16.8 ± 0.78. The female only laid eggs once in her life and died within 7 d after the end of laying.

### 3.5 Description of rivalry behavior

When multiple males were on the same leaf, two types of interference signals, DPs or MDSs, could be detected in the absence of females, indicating that the rivalry behavior among males was triggered by the MCaS. We detected DP signals during trials with two males (2/15) or two males and one female (5/29), while DPs or MDSs were detected in trials with four males (DP: 3/15, MDS 2/15) or two females and two males (DP: 6/16, MDS 3/16).

**Figure 7:**
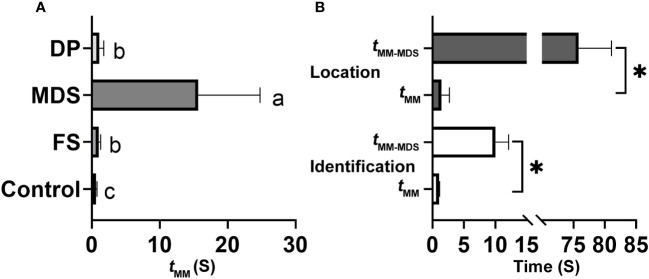
Effect of different signals on the interval between two neighboring male calling signals (MCaSs) in male *Empoasca onukii.* **(A)** Effect of inserted female signal (FS), mating disruption signal (MDS) and disruptive pulses (DP) on the value of *t*_MM_ during the identification stage, where *t*_MM_ is the interval from the end of an MCaS to the beginning of the next neighboring MCaS. **(B)** Effect of MDS playback on the value of *t*_MM_ during identification (down) and location stage (up). Mean values with different letters or * indicate a significant difference within a histogram (*P* < 0.05, paired *t*-test).

When the rival detected an MCaS from another male, it employed two types of rivalrous strategies: (1) following the second MCaS, the rival emitted MDSs to form competing duets in an MCaS-MDS-MCaS-MDS sequence. Each time the rival emitted an MDS, the male failed to enter the location stage and re-entered the call-fly stage. Competing duets were only detected in the identification stage, not in the location stage. Compared with *t*_MM-FS_, MDSs significantly prolonged the interval of MCaSs (*t*_MM-MDS_ = 15.68 ± 9.1 s; *t* = 9.802, *df* = 4, *P* < 0.001; **Figure 7A**). During the competing duet, the female did not reply until MCaSs were detected without being followed by MDSs. The rival disrupted the identification duet of a pair of leafhoppers using MDSs, and then emitted its own MCaSs to establish mating communication with the female. (2) When the second MCaS was detected, the rival emitted DPs to overlap the MCaSs, during which the rival located the courting male and drove it away after contact. Some rivals emitted DPs to overlap MCoSs in a pair’s location stage; most DPs overlapped S1 of the MCaS or MCoS (**Figure 2D**), and a few overlapped S2. Courting males clearly sensed the presence of a nearby rival when its signal was overlapped by a DP, because *t*_MM-DP_ was significantly prolonged (*t* = 4.837, *df* = 4, *P* < 0.001) compared with the blank controls’ *t*_MM_ (**Figure 7A**). In only one trial, the male disturbed by the MDS tried to locate the rival, which had established an identification duet with the female, and the first male emitted DPs to overlap MCaSs from the rival.

In each trial, one MDS (4.48 s) playback blocked the leafhopper’s identification and location duet (15/15), and the male would re-enter the call-fly stage and emit the MCaS two to three times to re-establish the identification duet. Playback of one MDS significantly prolonged the identification duets (*t* = 9.322, *df* = 4, *P* = 0.001), and the intervals between adjacent MCaSs were extended from 1.03 ± 0.1 s (*t*_MM_) to 10 ± 2.12 s (*t*_MM-MDS_, **Figure 7B**). When the location duet was disrupted by the playback of one MDS, the male would emit the MCaS instead of the MCoS and conduct the identification process again. Therefore, the intervals were significantly extended to 75.84 ± 5.24 s (*t*_MM-MDS_), compared with those in the control (*t*_MM_ = 1.43 ± 1.26 s; *t* = 30.579, *df* = 4, *P* < 0.001; **Figure 7B**). Because no real rival appeared, all male-female pairings (15/15) were successfully completed during playback of the DP, and there was no significant difference between *t*_MM_ and *t*_MM-MDS_ (*P* > 0.05), i.e., the playback of DPs had no effect on the mating duet or the ratio of female responses to male calling.

## 4 Discussion

Gordon and Krugner (2019) have proposed a step-by-step method for creating effective synthetic vibrational signals for playback to disrupt the mating communication of *H. vitripennis*. The first step is to describe the basic communication behaviors of the pest. The second step is to identify which candidate signals disrupt communication. Finally, execution tests are conducted to determine which signals effectively disrupt mating in the laboratory and the field.

Besides effective disruptive signals, accurate investigations of the biology and physiology of the pest are required to define the time of device application during the 24-h period and the amplitude threshold of efficacy of the disruptive signals (Polajnar et al., 2016). These practical issues are important for optimizing energy consumption and signal transmission to ensure the efficacy of the VMD (Mazzoni et al., 2019).

### 4.1 Mating behaviors

The narrow time window of mating activity of pests is conducive to shortening the time of location and therefore reducing the risk of predation (Virant-doberlet et al., 2011). Like *S. titanus* (Mazzoni et al., 2009b) and *M. pruinosa* (Virant-doberlet and Žežlina, 2007), the mating activity of *E. onukii* had an obvious narrow time window and was restricted to the periods of dawn and dusk, which was highly consistent with its flight activity (Bian et al., 2014). The temporal distribution of mating activity of *E. onukii* is helpful for setting the time of VMD application. However, some species could adjust their temporal calling pattern to overcome external adverse factors (McNett et al., 2010). Whether *E. onukii* has this ability still needs further research.

For males and females, the mating of *E. onukii* was concentrated on the sixth or seventh mature leaf below the tea bud. The spatial proximity of male and female *E. onukii* did not shorten the location time of males, so their spatial distribution pattern is more likely to facilitate signal transmission and perception. Compared with young leaves, mature leaves have a larger surface area and greater hardness, which may be more advantageous to *E. onukii* signal transmission (Michelsen et al., 1982). When the distance between the male and female is relatively large, insects such as stinkbugs can use pheromones to gather in multi-modal communication systems (Čokl et al., 2019). So far, no chemical substance has been reported in the mating behavior of leafhoppers (Cocroft, 2011), so a long spatial distance may not be conducive for mating communication of leafhoppers (Ord and Stamps, 2008). In this study, when the distance between the male and female was more than three leaves, the probability of a female response to the MCaS decreased, which may be because the distance was too far for the female to detect the MCaS. In other words, the signal amplitude was close to or below the female’s perception threshold.

It is necessary to consider the effect of host complexity on the mating behavior of pests. The vibrational signal will form a complex active network on the plant (Mazzoni et al., 2014). When the experimental condition changes from a single leaf to a branch, the leafhoppers’ behavior and signals may change. For example, on a single vine leaf, the *S. titanus* male calling song turns into a courtship song immediately after the first female reply (Mazzoni et al., 2009b). When males and females are placed on two different leaves on the same grapevine shoot, after the female reply, the mating process proceeded differently, including the type of signals and behaviors that the males subsequently exhibited (Mazzoni et al., 2014; Polajnar et al., 2016). When males and females are confined to the same leaf, the intensity of signals will increase because the distance between the male and female is shortened, and the intensity may be an important cue for insects to judge distance and direction (Michelsen et al., 1982). In this study, some *E. onukii* males moved to the adjacent stem immediately after emitting the MCaS, perhaps because the potential FS1 was more easily perceived on the stem.

### 4.2 Disruptive signals

Effective synthetic disruptive signals are one of the key requirements for developing VMD (Mazzoni et al., 2019). *Empoasca onukii* males naturally exhibited two distinct signal rival behaviors, primarily expressed by the emission of disruptive signals, the DP and MDS. Unlike the DP of *E. vitis* (Nieri and Mazzoni, 2018) and *Tylopelta gibbera* (Legendre et al., 2012), *E. onukii* DPs had no interrupting effect on the ongoing duet or the ratio of female responses to male calling. Behaviorally, *E. onukii*’s DP seemed like a defiant signal, where the rival located the males and engaged in physical conflict to drive them away. Compared with DPs, MDSs of male *E. onukii* could effectively interrupt the ongoing identification duets and prevent courting leafhoppers from entering the location stage. This strategy was also found in the males of *S. titanus*, which disrupted the ongoing duet and prevented the leafhoppers from entering the proper courtship phase by emitting disturbance noise (Mazzoni et al., 2009b). In addition, the rivals of some leafhopper species, including *S. titanus* (Mazzoni et al., 2009b) and *A. makarovi* (Kuhelj and Virant-Doberlet, 2017), can obtain preferential copulating by eavesdropping on the female’s response signals and silently approaching the female. We suspected that rivals of *E. onukii* also had this ability to approach the female while sending DPs to disturb males (but without emitting either the MCaS or MCoS to alert the female to their presence). However, this assumption was rejected, because even in the absence of females, rivals still emitted DPs and drove other males away.

MDS can block 100% of ongoing identification and location duets on the first playback, which makes it a suitable choice for future VMD of *E. onukii* in the field. However, the male-female identification duets maybe re-established immediately after MDS playback stops, and this phenomenon is likely to be the result of a behavior referred to as gap detection (McNett et al., 2010). During the entire rivalry process, courting males and rivals followed a precise temporal pattern and exchanged information in real time (Polajnar et al., 2014). Playing back the MDS to interrupt *E. onukii*’s MCaS requires strict synchronization on the millisecond scale, which is extremely difficult to achieve (Mankin et al., 2013; Korinšek et al., 2016). However, if the MDS was played back continuously, it may cause *E. onukii* to rapidly habituate (Polajnar et al., 2015). Because of the significant differences of signal parameters among *E. onukii* individuals, habituation may be avoided by creating disruptive signals with real-time parameter changes. It has been reported that constantly changing the parameters of the disruptive signals may avoid the rapid habituation of the disturbed targets (Gilsdorf et al., 2003). Regardless, avoiding habituation of *E. onukii* to disruptive signals is one of the goals of our future research.

### 4.3 Application assumption

Light traps and colored sticky boards are commonly used to trap and kill *E. onukii* in the field (Bian et al., 2018b; Bian et al., 2021). However, although the proportion of males captured by either of these methods was high, the control effect was limited. *Empoasca o*nukii have a strong reproductive capacity, and males are known to mate multiple times. In principle, it is necessary to remove a very high proportion of males from a pest population to achieve a significant effect on subsequent generations (Polajnar et al., 2019). In fact, the strategy of ‘attraction and trapping’ is restricted to the searching/flying activity of the target pest (Polajnar et al., 2014). Recently, VMD has been found to increase the flight activity of *S. titanus* (Zaffaroni-Caorsi et al., 2022). In view of *E. onukii* re-entering the call-fly stage after interruption by the MDS, the combination of ‘attraction and trapping’ and VMD may further improve the control effectiveness of *E. onukii*, and lead to the development of a novel method for integrated pest management of this important tea pest.

In conclusion, this study demonstrated that researching the mating behavior of tea leafhoppers on a complex host is necessary. During their mating behavior, *E. onukii* aggregate in time and space. This knowledge can facilitate the energy-efficient application of VMD technology in the field by setting the devices to emit disruptive signals at the optimal times for this species. Additionally, the amplitude of disruptive signals on mature leaves should be higher than the perception threshold of *E. onukii* to ensure the efficacy of the technology. MDSs can be used to disrupt the mating of *E. onukii*, but further studies are needed to optimize the signal parameters or technical application methods to avoid the habituation of *E. onukii* after continuous interference. Admittedly, there are limitations to the findings of our study. The effects of disruptive signals on the mating, spawning, and reproductive rate of *E. onukii* still need to be tested both indoors and outdoors, and host complexity and environmental factors must be considered. Moreover, unlike the mating interference of leafhoppers on grapes, air is the only alternative signal transmission medium for *E. onukii* in tea gardens. Future studies need to consider how to develop playback devices to transmit disruptive signals to the host through the air, as well as avoiding environmental pollution, reducing costs, and expanding effective coverage.

## Data availability statement

The raw data supporting the conclusions of this article will be made available by the authors, without undue reservation.

## Ethics statement

The manuscript presents research on animals that do not require ethical approval for their study.

## Author contributions

LB: Conceptualization, Data curation, Formal Analysis, Funding acquisition, Methodology, Project administration, Resources, Supervision, Validation, Writing – original draft, Writing – review & editing. YS: Formal Analysis, Investigation, Validation, Writing – original draft. X-SZ: Formal Analysis, Investigation, Validation, Writing – original draft. X-MC: Data curation, Project administration, Resources, Supervision, Writing – review & editing. Z-XL: Data curation, Formal Analysis, Resources, Writing – review & editing. Z-QL: Data curation, Formal Analysis, Resources, Writing – review & editing. C-LX: Data curation, Formal Analysis, Resources, Writing – review & editing. Z-MC: Conceptualization, Formal Analysis, Project administration, Resources, Supervision, Writing – review & editing.
