# Supplementary material for: Mating and post-copulation behavior in the tea leafhopper, Empoasca onukii (Hemiptera: Cicadellidae)
Source: Front Plant Sci. 2023 Oct 4;14:1273718. doi: 10.3389/fpls.2023.1273718 (PMC10583563; doi:10.3389/fpls.2023.1273718)
Supplement: Supplementary file 1 [file Table_1.docx]

**Table S1** Spectral and temporal parameters of vibrational signals from a single *Empoasca onukii* female.

|  | *Df*_b_ (Hz) | *Df*_e_ (Hz) | *MR* (Hz/ms) | Duration (s) | Intensity (mm/s) |
| --- | --- | --- | --- | --- | --- |
| FS1 | 510.34 ± 5.9 | 202.41 ± 19.26 | -1.537 ± 0.251 | 0.233 ± 0.029 | 0.039 ± 0.003 |
| FS2 | 475.88 ± 14.04 | 411.28 ± 9.01 | -0.837 ± 0.056 | 0.118 ± 0.015 | 0.027 ± 0.002 |
| *t* | 5.06 | 21.963 | 6.097 | 7.906 | 6.203 |
| *P* | 0.001 | 0.001 | 0.003 | < 0.001 | 0.003 |

FS1, female signal response to the male call signal; FS2, female signal response to the male courtship signal; *Df*_b_, starting dominant frequency; *Df*_e_, ending dominant frequency; *MR*, modulation rate determining the *Df* rate of increase/decrease within a signal. Significant differences in the signal parameters between FS1 and FS2 were analyzed using an paired *t*-test (*P* < 0.05).
