# Supplementary material for: Mating and post-copulation behavior in the tea leafhopper, Empoasca onukii (Hemiptera: Cicadellidae)
Source: Front Plant Sci. 2023 Oct 4;14:1273718. doi: 10.3389/fpls.2023.1273718 (PMC10583563; doi:10.3389/fpls.2023.1273718)
Supplement: Supplementary file 2 [file Table_2.docx]

**Table S2** Standardized canonical discriminant function coefficients.

|  | Function 1 | Function 2 |
| --- | --- | --- |
| *Df*_b_ | 0.722 | 0.631 |
| *Df*_e_ | -0.207 | 0.183 |
| *MR* | 1.115 | -1.291 |
| Duration/*PRT* | -1.195 | 1.076 |
