# Supplementary material for: Mating and post-copulation behavior in the tea leafhopper, Empoasca onukii (Hemiptera: Cicadellidae)
Source: Front Plant Sci. 2023 Oct 4;14:1273718. doi: 10.3389/fpls.2023.1273718 (PMC10583563; doi:10.3389/fpls.2023.1273718)
Supplement: Supplementary file 3 [file Table_3.docx]

**Table S3** Classification results (%) from discriminant analysis.

| Original type | Predicted signal type | | | | | | |
| --- | --- | --- | --- | --- | --- | --- | --- |
|  | FS1 | FS2 | DP | MCaS-S0 | MCaS-S1 | MCaS-S2 | MDS |
| FS1 | **100.0** | 0.0 | 0.0 | 0.0 | 0.0 | 0.0 | 0.0 |
| FS2 | 0.0 | **80.0** | 0.0 | 0.0 | 0.0 | 0.0 | 20.0 |
| DP | 0.0 | 0.0 | **100.0** | 0.0 | 0.0 | 0.0 | 0.0 |
| MCaS-S0 | 0.0 | 0.0 | 0.0 | **100.0** | 0.0 | 0.0 | 0.0 |
| MCaS-S1 | 0.0 | 0.0 | 0.0 | 0.0 | **100.0** | 0.0 | 0.0 |
| MCaS-S2 | 0.0 | 0.0 | 0.0 | 20.0 | 0.0 | **80.0** | 0.0 |
| MDS | 0.0 | 0.0 | 0.0 | 0.0 | 0.0 | 0.0 | **100.0** |

Male and female vibrational signals were assigned to a certain type of signal based on temporal (duration or pulse repetition time) and spectral (starting dominant frequency, ending dominant frequency and modulation rate) features of the signal. Percentages in bold font represent signals that were correctly assigned to the signal type. FS1, female signal response to male calling signal; FS2, female signal response to male courtship signal; DP, disruptive pulse; MCaS-S0, MCaS-S1, and MCaS-S2 represent the first three sections of the male calling signal (MCaS); MDS, mating disruption signal.
